# Supplementary figures and images for: Isolation and characterization of halophilic and halotolerant fungi from man-made solar salterns in Pattani Province, Thailand
Source: PLoS One. 2023 Feb 13;18(2):e0281623. doi: 10.1371/journal.pone.0281623 (PMC9925087; doi:10.1371/journal.pone.0281623)

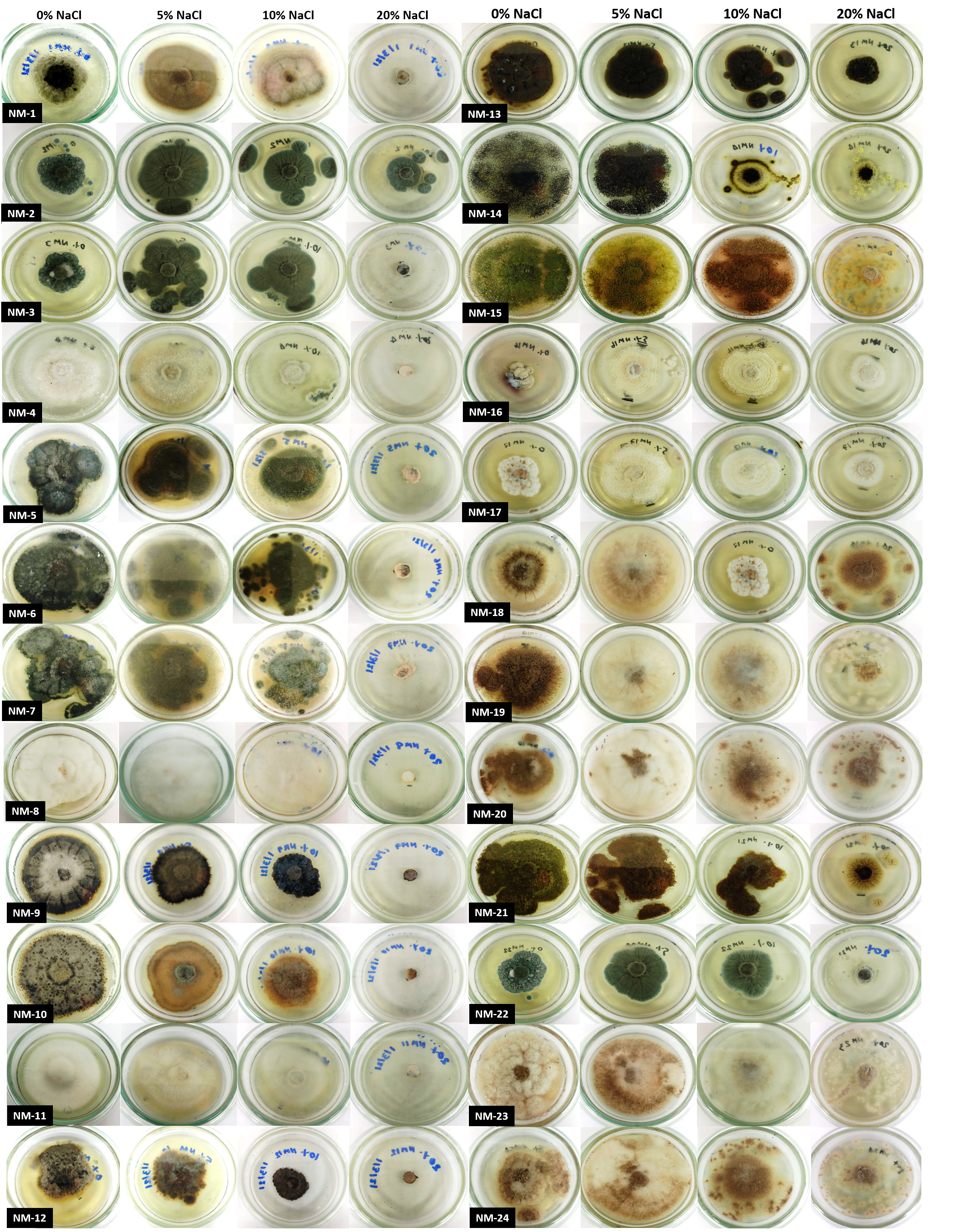

Supplement: S1 Fig — Results were evaluated based on colony diameter after 14 days of growth at 28°C under various NaCl concentrations (0–20%(w/v)). (TIF) [file pone.0281623.s001.tif]
