# Supplementary material for: Isolation and characterization of halophilic and halotolerant fungi from man-made solar salterns in Pattani Province, Thailand
Source: PLoS One. 2023 Feb 13;18(2):e0281623. doi: 10.1371/journal.pone.0281623 (PMC9925087; doi:10.1371/journal.pone.0281623)
Supplement: S1 Table — Antimicrobial activity of ethanolic extracts of the culture broths and mycelia of eight active isolates using the agar well diffusion method. (PDF) [file pone.0281623.s002.pdf]

Ethanolic extracts of the fungal culture broths

	Test organism	NM-3 1	NM-3 10	NM-3 100	NM-8 1	NM-8 10	NM-8 100	NM-15 1	NM-15 10	NM-15 100	NM-18 1	NM-18 10	NM-18 100	(+)ve control (20 µg/ml)
		**Concentration of the extract (mg/ml)**												
Zone of inhibition (mm)	EF*	-	-	31.6±2.4	-	19.9±0.3	28.7±0.3	-	-	14.7±0.9	-	20.8±0.3	33.6±0.5	13.7±1.9
	ML	19.9±0.8	24.9±1.0	39.3±1.4	20.5±0.3	26.4±1.1	29.3±0.2	-	-	19.8±0.2	-	10.8±0.6	22.9±0.6	18.5±0.1
	SA	12.7±0.6	19.5±1.1	28.1±1.3	18.4±0.7	22.8±1.0	23.5±1.2	-	-	13.6±0.5	-	16.0±0.0	30.0±1.9	14.7±0.5
	SE	14.3±0.1	22.9±2.3	33.9±0.9	21.1±0.0	26.2±0.2	28.1±0.5	-	-	16.1±0.6	-	16.5±0.7	25.1±0.6	13.1±0.6
	MRSA	14.9±0.4	20.9±0.4	31.8±0.6	19.2±0.6	25.7±0.6	26.6±0.4	-	-	16.0±0.2	-	14.1±0.3	28.0±2.3	12.9±0.3
	EC	-	-	-	-	-	-	-	12.2±0.2	23.6±0.0	-	14.5±0.9	27.2±0.8	19.7±1.7
	PA	-	-	-	-	-	-	-	-	21.7±0.9	-	-	-	14.2±0.5
	ST	24.5±0.0	27.9±0.7	41.9±0.3	21.6±0.9	28.4±0.7	31.1±1.8	-	-	24.7±2.5	-	18.7±0.0	30.3±0.7	20.7±1.2
	CA	-	18.8±1.6	36.2±2.4	10.9±0.4	15.9±0.4	16.4±0.3	-	-	-	-	-	29.6±0.4	19.3±0.6
	AF	-	-	20.5±0.5	-	13.1±1.3	20.9±0.0	-	-	13.1±0.1	-	-	20.5±1.5	13.9±1.9
	MC	-	-	-	-	-	12.7±0.3	-	-	-	-	-	-	12.6±0.3

Ethanolic extracts of the fungal mycelia

	Test organism	NM-3 1	NM-3 10	NM-3 100	NM-8 1	NM-8 10	NM-8 100	NM-15 1	NM-15 10	NM-15 100	NM-18 1	NM-18 10	NM-18 100	(+)ve control (20 µg/ml)
		**Concentration of the extract (mg/ml)**												
Zone of inhibition (mm)	EF	-	-	24.1±1.2	12.9±0.0	20.9±0.3	21.9±0.4	-	-	-	-	-	29.3±2.0	13.7±1.9
	ML	-	11.3±0.2	24.5±2.6	13.5±0.2	20.9±0.9	23.4±0.8	-	-	16.0±0.3	-	-	20.1±0.5	18.5±0.1
	SA	-	10.3±0.8	17.5±0.7	17.5±0.1	22.9±0.2	23.3±0.2	-	-	16.6±0.3	-	-	15.1±0.4	14.7±0.5
	SE	-	10.7±0.6	23.1±0.0	15.6±0.1	20.2±0.3	22.2±1.7	-	-	10.9±0.2	-	-	14.3±0.1	13.1±0.6
	MRSA	10.5±0.6	13.2±0.9	20.4±0.5	16.5±0.4	21.4±1.5	23.6±0.5	-	-	12.0±0.5	-	-	12.3±0.0	12.9±0.3
	EC	-	-	-	-	-	18.3±0.3	-	-	16.7±0.6	-	-	13.5±0.7	19.7±1.7
	PA	-	-	-	-	-	-	-	-	13.6±0.5	-	-	10.6±0.6	14.2±0.5
	ST	11.9±0.4	16.1±0.0	30.9±0.4	14.5±0.0	22.3±0.9	24.4±1.6	-	-	-	-	-	19.4±1.2	20.7±1.2
	CA	-	-	20.5±0.0	-	-	-	-	-	-	-	-	-	19.3±0.6
	AF	-	-	-	-	-	15.5±0.2	-	-	-	-	-	-	13.9±1.9
	MC	-	-	-	-	-	-	-	-	-	-	-	-	12.6±0.3

Ethanolic extracts of the fungal culture broths

Zone of inhibition (mm)	Test organism	NM-19			NM-20			NM-23			NM-24			(+)ve control (20 µg/ml)
		**Concentration of the extract (mg/ml)**												
		1	10	100	1	10	100	1	10	100	1	10	100	
	**EF**	-	20.3±0.9	39.7±1.7	-	21.1±0.2	38.3±1.6	-	18.9±0.7	38.5±2.0	-	20.8±0.2	35.3±1.6	13.7±1.9
	**ML**	-	10.4±1.8	29.1±0.4	-	12.0±0.3	31.4±1.7	-	11.9±0.1	29.9±0.4	-	11.6±0.3	31.4±1.2	18.5±0.1
	**SA**	-	19.3±1.1	26.3±0.4	-	20.2±0.2	30.4±0.3	-	19.5±0.2	29.6±0.7	-	18.4±0.3	29.5±0.8	14.7±0.5
	**SE**	-	12.9±0.0	29.6±0.5	-	12.6±0.5	32.9±0.8	-	14.9±0.4	31.3±0.9	-	13.9±0.6	31.5±0.5	13.1±0.6
	**MRSA**	-	14.1±0.6	27.5±1.0	-	20.2±0.3	30.9±0.9	-	14.2±1.0	30.2±1.7	-	14.3±0.8	29.3±0.2	12.9±0.3
	**EC**	-	16.0±0.4	29.0±0.2	-	16.6±0.0	29.6±1.1	-	13.2±0.3	29.3±2.4	-	13.6±0.0	31.1±0.2	19.7±1.7
	**PA**	-	-	14.6±0.5	-	-	12.4±0.4	-	-	11.6±0.0	-	-	12.5±0.2	14.2±0.5
	**ST**	-	20.2±0.8	32.1±0.2	-	20.6±0.3	29.6±1.8	-	18.9±0.7	31.9±0.3	-	20.0±0.5	32.6±0.9	20.7±1.2
	**CA**	-	-	28.5±0.7	-	-	27.1±0.7	-	12.2±0.0	27.3±0.7	-	12.6±0.4	31.0±0.7	19.3±0.6
	**AF**	-	-	23.5±1.1	-	-	22.6±0.9	-	-	22.1±1.1	-	-	24.1±1.1	13.9±1.9
	**MC**	-	-	-	-	-	-	-	-	15.9±0.4	-	-	16.2±0.4	12.6±0.3

Ethanolic extracts of the fungal mycelia

Zone of inhibition (mm)	Test organism	NM-19			NM-20			NM-23			NM-24			(+)ve control (20 µg/ml)
		**Concentration of the extract (mg/ml)**												
		1	10	100	1	10	100	1	10	100	1	10	100	
	**EF**	-	-	24.8±0.8	-	-	28.2±0.4	-	-	29.8±0.4	-	-	30.9±0.5	13.7±1.9
	**ML**	-	-	11.6±0.3	-	-	17.1±1.0	-	-	16.5±0.9	-	-	17.1±0.7	18.5±0.1
	**SA**	-	-	10.6±0.2	-	-	10.3±0.2	-	-	10.1±0.0	-	-	11.0±0.1	14.7±0.5
	**SE**	9.1±0.2	12.6±0.4	18.5±0.4	-	-	16.7±0.8	-	-	14.1±0.3	-	-	13.1±0.9	13.1±0.6
	**MRSA**	-	-	-	-	-	-	-	-	9.7±0.0	-	-	-	12.9±0.3
	**EC**	-	-	11.6±0.2	-	-	11.6±0.2	-	-	11.9±0.3	-	-	8.8±0.0	19.7±1.7
	**PA**	-	-	-	-	-	-	-	-	-	-	-	-	14.2±0.5
	**ST**	-	-	17.6±0.5	-	-	17.6±0.2	-	13.9±0.4	16.4±1.5	-	13.1±0.3	16.7±0.2	20.7±1.2
	**CA**	-	-	-	-	-	-	-	16.2±0.5	23.0±0.4	-	-	-	19.3±0.6
	**AF**	-	-	-	-	-	-	-	-	-	-	-	-	13.9±1.9
	**MC**	-	-	-	-	-	-	-	-	-	-	-	-	12.6±0.3

*EF: *Enterococcus faecalis*; ML: *Micrococcus luteus* (ATCC9341); SA: *Staphylococcus aureus* (ATCC25923); SE: *S. epidermidis*; MRSA: methicillin-resistant *S. aureus* (MRSA), EC: *Escherichia coli* (ATCC25922), PA: *Pseudomonas aeruginosa* (ATCC27853); ST: *Salmonella* Typhi (ATCC19430); CA: *Candida albicans* (ATCC90028), AF: *Aspergillus fumigatus* (AF293); and MC: *Microsporum canis*. Vancomycin and gentamicin (20 µg/ml) were used as standard antibacterial agents. Amphotericin B and miconazole (20 µg/ml) were used as standard antifungal agents. -: no activity.
