# Supplementary material for: Isolation and characterization of halophilic and halotolerant fungi from man-made solar salterns in Pattani Province, Thailand
Source: PLoS One. 2023 Feb 13;18(2):e0281623. doi: 10.1371/journal.pone.0281623 (PMC9925087; doi:10.1371/journal.pone.0281623)
Supplement: S1 Raw image — (PDF) [file pone.0281623.s003.pdf]

Original gel image

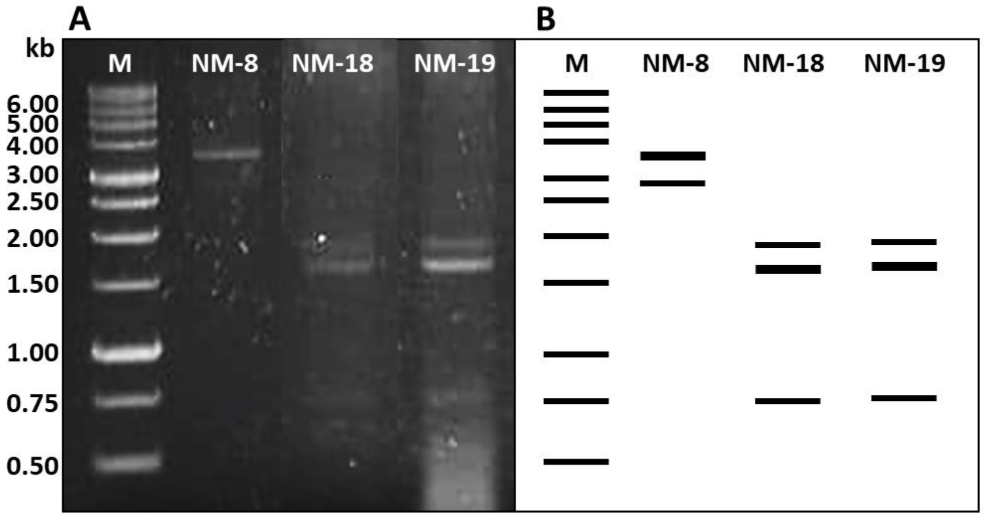

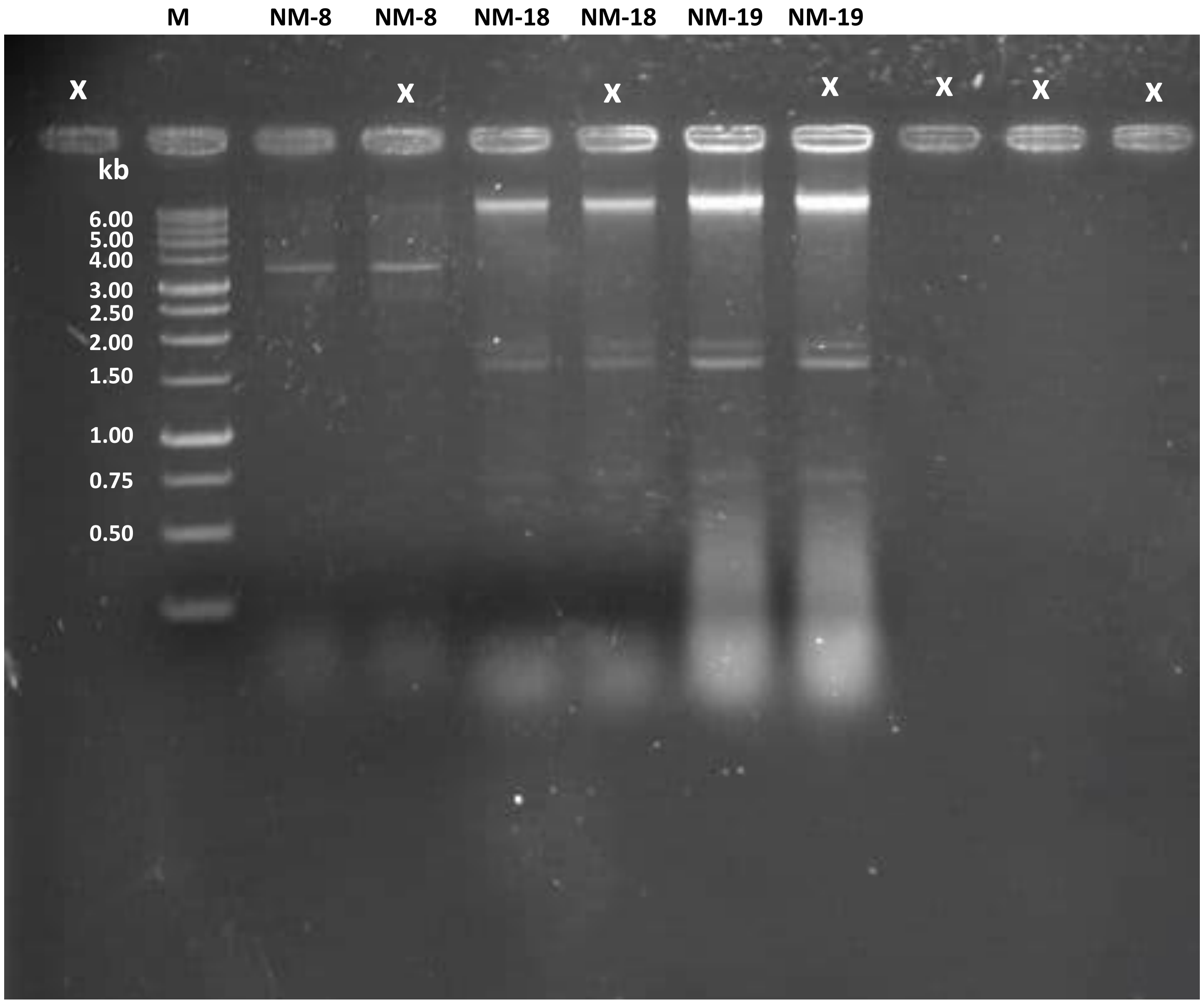

Note: duplicate lanes of each sample and un-used lanes were cropped to generate Fig. 5.
