# Supplementary material for: Isolation and characterization of halophilic and halotolerant fungi from man-made solar salterns in Pattani Province, Thailand
Source: PLoS One. 2023 Feb 13;18(2):e0281623. doi: 10.1371/journal.pone.0281623 (PMC9925087; doi:10.1371/journal.pone.0281623)
Supplement: Attachment — Submitted filename: Response to Reviewers.docx [file pone.0281623.s004.docx]

**Department of Microbiology, Faculty of Science,**

**Prince of Songkla University, Hat Yai, 90110 Thailand**

29^th^ November 2022

Dear Editor,

With reference to the above manuscript, the revised version we are submitting to PLOS ONE for publication. We have answered all of your queries re the manuscript and would like to take this opportunity to thank you and the referees for guiding us through the process of submitting our work for publication in the journal. Our comments to all queries are shown in blue typeface. We have also provided the original underlying image for gel data reported in our submission and it has been uploaded as a Supporting Information file.

Needless to say, if there are any further questions or queries re the manuscript do not hesitate to get back in contact with me.

Yours sincerely,

Lakkhana Kanhayuwa Wingfield

Division of Biological Science, Faculty of Science,

Prince of Songkla University, Hat Yai, 90112 Thailand

Email: Lakkhana.k@psu.ac.th

**Reviewers' comments:**

Reviewer's Responses to Questions

**Comments to the Author**

1. Is the manuscript technically sound, and do the data support the conclusions?

The manuscript must describe a technically sound piece of scientific research with data that supports the conclusions. Experiments must have been conducted rigorously, with appropriate controls, replication, and sample sizes. The conclusions must be drawn appropriately based on the data presented.

Reviewer #1: Yes

Reviewer #2: Yes

2. Has the statistical analysis been performed appropriately and rigorously?

Reviewer #1: Yes

Reviewer #2: Yes

3. Have the authors made all data underlying the findings in their manuscript fully available?

The PLOS Data policy requires authors to make all data underlying the findings described in their manuscript fully available without restriction, with rare exception (please refer to the Data Availability Statement in the manuscript PDF file). The data should be provided as part of the manuscript or its supporting information, or deposited to a public repository. For example, in addition to summary statistics, the data points behind means, medians and variance measures should be available. If there are restrictions on publicly sharing data—e.g., participant privacy or use of data from a third party—those must be specified.

Reviewer #1: Yes

Reviewer #2: Yes

4. Is the manuscript presented in an intelligible fashion and written in standard English?

PLOS ONE does not copyedit accepted manuscripts, so the language in submitted articles must be clear, correct, and unambiguous. Any typographical or grammatical errors should be corrected at revision, so please note any specific errors here.

Reviewer #1: No

Reviewer #2: No

**5. Review Comments to the Author**

**Reviewer #1:**

1. The overall English of the manuscript needs extensive revision and improvement.

\## The proposed revision has been made.

2. The literature review in introduction as well as paper comparisons with previous publications are citing decade old citations which better needs to be from last 5 years or at most from 7-8 years older. Specifically, the halophilic fungal diversity and applications needs to be updated.

\## I understand that some citations presented in the literature review and discussion of this manuscript are not very up-to-date. After reviewing and researching for more recent publications (2018-2022) on halophilic fungi, most of the recent works have been focused on cell biology, physiological response (particularly stress response, and mechanisms of cellular and molecular adaptation to salinity), and in-depth investigation on metabolic potential of halophilic fungi. We assume they are rather irrelevant to our research scope which focuses on fungal diversity and their biotechnological potential (initial investigation).

We also try to cover the most current research in the field of halophilic fungal diversity by including both early and very new research. However, few recent publications have described fungal diversity in hypersaline environments, for example; Chamekh et al., 2019. (Isolation, identification and enzymatic activity of halotolerant and halophilic fungi from the Great Sebkha of Oran in northwestern of Algeria. Mycobiology. 47(2):230-241), Rashmi *et al*., 2018. (Fungal diversity of the hypersaline Inland Sea in Qatar. Botanica Marina. 61(6), 595-609., Chung et al., 2019. (Fungi in salterns. J Microbiol. 57(9), 717-724) and Dendouga et al., 2022. (Screening of halotolerant microfungi isolated from hypersaline soils of Algerian Sahara for production of hydrolytic enzymes. J of Biol Res. 95:10167). In addition, up-to-date publication on antimicrobial activity of *Penicillium* was published (Orfali et al. Anti-microbial activity of dihydroisocoumarin isolated from Wadi Lajab sediment-derived fungus *Penicillium* *chrysogenum*: *In vitro* and *In silico* study. Molecules. 2022; 27: 3630. https://doi.org/10.3390/molecules27113630). These publications are cited in our manuscripts (## Line 367-670, 403-408, 434-436).

As suggested, more recent citations would strengthen the presentation of the manuscript. I strongly believe this logic. However, as far as I am concerned, some recent publications on halophilic fungi still cite old publications in order to support their findings/hypotheses, for example, Pupo *et al*., 2021. (Osmolyte signatures for the protection of *Aspergillus sydowii* cells under halophilic conditions and osmotic shock. J Fungi. 7, 414. https://doi.org/10.3390/jof7060414) as well as publications aforementioned.

In our case, some old references are used because they need to be able to cover the materials. We cited old publications on a case-by-case basis. For example, the methods we followed belong to the original publications which remain applicable nowadays (identification keys of fungi, primer set for PCR amplification, enzyme and antimicrobial assays). Result interpretation, for example, enzyme activity was evaluated by an enzymatic index described by original work of Hankin (900 citations).

In term of diversity of fungal halophiles (either stated on introduction or discussion), we sometimes compare our results or cite literature to decade-old publications because they are relevant to the point we are trying to make. For example, we demonstrated fungal genera found from our sampling sites. These included *A. subalbidus* and *Fusarium* which were also reported from two old publications (2006 and 2014). In literature review, we include old citations to introduce fungal genera that have been found from hypersaline environments so far. Similarly, description of characteristics of hypersaline soils, groups of halophiles, stress response to salinity in fungi we cited here were from the original publications. Overall, in my opinion, much more relevant than publication year is the content of the publications cited that cover relevant literature/discussion. We definitely try to cover the most current research in the field of halophilic fungal diversity by including both early and very new research as stated. However, if you have any additional suggestion regarding this matter, I would be very appreciated to correct points you suggest.

3. Try to discuss that how these fungi fall in halophilic category by consulting publications such as Hypersaline habitats and halophilic microorganisms, 2016, Maejo International Journal of Science and Technology 10(3):330-345.

\## The proposed correction has been made in the revised version of our manuscript.

4. For the unit of ml or ul, use capital L.

\## The proposed corrections have been made in the revised version of our manuscript.

5. Better add a separate conclusion having future perspective of your research.

\## The proposed correction has been made in the revised version of our manuscript (line 442-454).

**Reviewer #2:** General

The manuscript requires a major rewriting to upgrade the quality of the English language presentation.

\## The proposed correction has been made in the revised version of our manuscript.

The citations in many instances for very old. More recent citations would strengthen the presentation of the manuscript.

\## I understand that some citations presented in the literature review and discussion of this manuscript are not very up-to-date. After reviewing and researching for more recent publications (2018-2022) on halophilic fungi, most of the research works have been focused on cell biology, physiological response (particularly stress response, and mechanisms of cellular and molecular adaptation to salinity), and in-depth investigation on metabolic potential of halophilic fungi. We assume they are rather irrelevant to our scope of research which focuses on fungal diversity and their biotechnological potential (initial investigation).

We also try to cover the most current research in the field of halophilic fungal diversity by including both early and very new research. However, few recent publications have described fungal diversity in hypersaline environments, for example; Chamekh et al., 2019. (Isolation, identification and enzymatic activity of halotolerant and halophilic fungi from the Great Sebkha of Oran in northwestern of Algeria. Mycobiology. 47(2):230-241), Rashmi *et al*., 2018. (Fungal diversity of the hypersaline Inland Sea in Qatar. Botanica Marina. 61(6), 595-609., Chung et al., 2019. (Fungi in salterns. J Microbiol. 57(9), 717-724) and Dendouga et al., 2022. (Screening of halotolerant microfungi isolated from hypersaline soils of Algerian Sahara for production of hydrolytic enzymes. J of Biol Res. 95:10167). In addition, up-to-date publication on antimicrobial activity of *Penicillium* was published (Orfali et al. Anti-microbial activity of dihydroisocoumarin isolated from Wadi Lajab sediment-derived fungus *Penicillium* *chrysogenum*: *In vitro* and *In silico* study. Molecules. 2022; 27: 3630. https://doi.org/10.3390/molecules27113630). These publications are cited in our manuscripts (## Line 367-670, 403-408, 434-436).

As suggested, more recent citations would strengthen the presentation of the manuscript. I strongly believe this logic. However, as far as I am concerned, some recent publications on halophilic fungi still cite old publications in order to support their findings/hypotheses, for example, Pupo *et al*., 2021. (Osmolyte signatures for the protection of *Aspergillus sydowii* cells under halophilic conditions and osmotic shock. J Fungi. 7, 414. https://doi.org/10.3390/jof7060414) as well as publications aforementioned.

In our case, some old references are used because they need to be able to cover the materials. We cited old publications on a case-by-case basis. For example, the methods we followed belong to the original publications which remain applicable nowadays (identification keys of fungi, primer set for PCR amplification, enzyme and antimicrobial assays). Result interpretation, for example, enzyme activity was evaluated by an enzymatic index described by original work of Hankin (900 citations).

In term of diversity of fungal halophiles (either stated on introduction or discussion), we sometimes compare our results or cite literature to decade-old publications because they are relevant to the point we are trying to make. For example, we demonstrated fungal genera found from our sampling sites. These included *A. subalbidus* and *Fusarium* which were also reported from two old publications (2006 and 2014). In literature review, we include old citations to introduce fungal genera that have been found from hypersaline environments so far. Similarly, description of characteristics of hypersaline soils, groups of halophiles, stress response to salinity in fungi we cited here were from the original publications which are undoubtedly old. Overall, in my opinion, much more relevant than publication year is the content of the publications cited that cover relevant literature/discussion. We definitely try to cover the most current research in the field of halophilic fungal diversity by including both early and very new research as stated. However, if you have any additional suggestion regarding this matter, I would be very appreciated to correct points you suggest.

For the first presentation of the genus and species of an organism, the genus and species should be spelled out. For subsequent occasions the first letter of the genus name can be abbreviated, i.e., Escherichia coli (first presentation) and E. coli (subsequent presentations).

\## The proposed corrections have been made in the revised version of our manuscript (line 26, 66, 173-176, 259-261, 266, 326-327 and discussion).

When using "et al." in the manuscript text or reference list, it should be in italics.

\## The proposed corrections have been made in the revised version of our manuscript (line 113, 313 and discussion).

Specific comments on the manuscript.

Pg. 4 line 94. The Fig. 1 legend requires more details. The windows should be labeled with an upper case letter designation (i.e. A, B,... ) and text should be provided in the legend to describe what is in each image.

\## The proposed corrections have been made in the revised version of our manuscript (lone 94).

Pg. 4 lines 101 and 102. The two analytical procedures require literature references as was done for the Tecator DS-6 Digester line 103.

\## The proposed corrections have been made in the revised version of our manuscript (line 101).

Pg. 7 line 163. Is the enzymatic index a standard approach to reporting a quantitative measure of enzyme activity. If so it requires a reference.

\## Yes, the enzymatic index stated in this manuscript is a standard approach to reporting a quantitative measure of enzyme activity as described by Hankin (1975).

The proposed correction has been made in the revised version of our manuscript (line 164).

Pg. 10 line 215. The Fig. 2 legend does not provide enough detail - see the line 94 comment on Fig. 1.

\## The proposed correction has been made in the revised version of our manuscript (line 212).

Pg. 15 line 318. The labes "A" and "B" are not indicated on Fig. 5.

\## The proposed correction has been made in the revised version of our manuscript (on Fig 5).

Pg. 16 line 354. Insufficient details is provided in the Fig. 6 legend.

\## The proposed correction has been made in the revised version of our manuscript (line 349).

Pg. 19 Line numbers are discontinued with the start of the Discussion section.

\## The proposed correction has been made in the revised version of our manuscript.

6. We note that the grant information you provided in the ‘Funding Information’ and ‘Financial Disclosure’ sections do not match. When you resubmit, please ensure that you provide the correct grant numbers for the awards you received for your study in the ‘Funding Information’ section.

\## The proposed correction has been made.

7. PLOS ONE now requires that authors provide the original uncropped and unadjusted images underlying all blot or gel results reported in a submission’s figures or Supporting Information files. This policy and the journal’s other requirements for blot/gel reporting and figure preparation are described in detail at https://journals.plos.org/plosone/s/figures#loc-blot-and-gel-reporting-requirements and https://journals.plos.org/plosone/s/figures#loc-preparing-figures-from-image-files. When you submit your revised manuscript, please ensure that your figures adhere fully to these guidelines and provide the original underlying images for all blot or gel data reported in your submission.

\## The proposed correction has been made.
