# Supplementary material for: Isolation and characterization of halophilic and halotolerant fungi from man-made solar salterns in Pattani Province, Thailand
Source: PLoS One. 2023 Feb 13;18(2):e0281623. doi: 10.1371/journal.pone.0281623 (PMC9925087; doi:10.1371/journal.pone.0281623)
Supplement: Attachment — Submitted filename: Response to Reviewers - 2.docx [file pone.0281623.s005.docx]

**Department of Microbiology, Faculty of Science,**

**Prince of Songkla University, Hat Yai, 90110 Thailand**

25^th^ January 2023

Dear Editor,

With reference to the above manuscript, the revised version we are submitting to PLOS ONE for publication. We have answered all of your queries re the manuscript and would like to take this opportunity to thank you and the referees for guiding us through the process of submitting our work for publication in the journal. Our comments and changes to all queries are shown in blue typeface. A rebuttal letter (labelled 'Response to Reviewers-2'), a marked-up copy of our manuscript (labelled 'Revised Manuscript with Track Changes-2') and an unmarked version of our revised paper (labelled 'Manuscript-1') are included along with submission.

Needless to say, if there are any further questions or queries re the manuscript do not hesitate to get back in contact with me.

Yours sincerely,

Lakkhana Kanhayuwa Wingfield

Division of Biological Science, Faculty of Science,

Prince of Songkla University, Hat Yai, 90112 Thailand

Email: Lakkhana.k@psu.ac.th

**Comments to the Author**

Journal Requirements:

Please review your reference list to ensure that it is complete and correct. If you have cited papers that have been retracted, please include the rationale for doing so in the manuscript text, or remove these references and replace them with relevant current references. Any changes to the reference list should be mentioned in the rebuttal letter that accompanies your revised manuscript. If you need to cite a retracted article, indicate the article’s retracted status in the References list and also include a citation and full reference for the retraction notice.

\# We have reviewed the reference list and no retracted paper was cited in our manuscript.

Additional Editor Comments (if provided):

Most of the concerns raised by the reviewers in their comments have been well address by the authors in their submitted revised manuscript. Two comments remain inadequately addressed, the manuscript would benefit by further enhancing the English presentation requested by both reviewers, and instances of improper usage of abbreviated genus names remain uncorrected in the manuscript. To assist in preparing a further revised version of the manuscript the following suggestions are offered for consideration by the authors. Line numbers used are those from the Track Changes version of the revised manuscript.

\# English presentation of our manuscript has been revised and improved by language editorials from the Publication Clinic Service at Prince of Songkla University. All changes are shown in blue typeface/highlight in our Revised Manuscript with Track Changes. Improper usage of abbreviated genus names was corrected as suggested. All suggestions were applied in our manuscript.
